# PPIMpred: a web server for high-throughput screening of small molecules targeting protein–protein interaction

**DOI:** 10.1098/rsos.160501

**Published:** 2017-04-19

**Authors:** Tanmoy Jana, Abhirupa Ghosh, Sukhen Das Mandal, Raja Banerjee, Sudipto Saha

**Affiliations:** 1 Bioinformatics Centre, Bose Institute, P 1/12, C.I.T. Road, Scheme-VII (M), Kolkata, West Bengal, India; 2 Department of Bioinformatics, Bose Institute, P 1/12, C.I.T. Road, Scheme-VII (M), Kolkata, West Bengal, India; 3 Department of Biotechnology, Maulana Abul Kalam Azad University of Technology, West Bengal, India

**Keywords:** protein–protein interaction, modulators, support vector machine, docking

## Abstract

PPIMpred is a web server that allows high-throughput screening of small molecules for targeting specific protein–protein interactions, namely Mdm2/P53, Bcl2/Bak and c-Myc/Max. Three different kernels of support vector machine (SVM), namely, linear, polynomial and radial basis function (RBF), and two other machine learning techniques including Naive Bayes and Random Forest were used to train the models. A fivefold cross-validation technique was used to measure the performance of these classifiers. The RBF kernel of SVM outperformed and/or was comparable with all other methods with accuracy values of 83%, 79% and 90% for Mdm2/P53, Bcl2/Bak and c-Myc/Max, respectively. About 80% of the predicted SVM scores of training/testing datasets from Mdm2/P53 and Bcl2/Bak have significant IC50 values and docking scores. The proposed models achieved an accuracy of 66–90% with blind sets. The three mentioned (Mdm2/P53, Bcl2/Bak and c-Myc/Max) proposed models were screened in a large dataset of 265 242 small chemicals from National Cancer Institute open database. To further realize the robustness of this approach, hits with high and random SVM scores were used for molecular docking in AutoDock Vina wherein the molecules with high and random predicted SVM scores yielded moderately significant docking scores (*p*-values < 0.1). In addition to the above-mentioned classification scheme, this web server also allows users to get the structural and chemical similarities with known chemical modulators or drug-like molecules based on Tanimoto coefficient similarity search algorithm. PPIMpred is freely available at http://bicresources.jcbose.ac.in/ssaha4/PPIMpred/.

## 1. Introduction

Protein–protein interactions (PPIs) play vital roles in several cellular processes, like signal transduction, cell proliferation, cell adhesion and apoptosis [1]. Many disease pathways including different stages of cancer development and host–pathogen interactions are associated with key PPIs [2]. Disruption of the crucially important PPIs is now thought to be a potential strategy to develop novel therapeutics [3]. Identification of hotspots at the interface or contact area of PPIs is now considered as a highly innovative and potential method to find newer drug targets [4–6]. The small chemical molecules that inhibit PPIs at their interfaces [7–9] are called PPI modulators (PPIMs). These PPIMs are very useful in designing drugs for various diseases including cancer. Though *in silico* identification of these compounds remains challenging in drug discovery, a few PPIMs have been identified and tested clinically in oncogenic studies. A few examples of small chemical PPIMs such as Nutlin-3a (Mdm2/P53) and ABT-263 and GX15-070 (Bcl2/Bak) were clinically tested [10–12]. Therefore, the interface areas of PPIs and identification of novel PPIMs which can inhibit an orthosteric region have been a central focus of many researchers.

In this study, three well-known oncogenic PPIs, namely Mdm2/P53, Bcl2/Bak and c-Myc/Max, were chosen as the model system for identifying novel PPIMs. These three PPIs are transient in nature and critically play roles in cell growth or programmed cell death (apoptosis), indicating their involvement in cell proliferation. Indeed, a plethora of studies had established their role in different stages of cancer development. Mdm2 is a negative regulator of P53, a tumour suppressor protein. P53 regulates cell cycle and induces apoptosis in response to various stresses, particularly DNA damage, thereby preventing or suppressing tumour progression and/or development [4,12]. Bcl2/Bak is a homologous PPI complex that has opposite effects on cell death and proliferation. Bcl2 helps in cell survival, and Bak has a vital role in accelerating programmed cell death. The c-Myc/Max complex is a nuclear phosphorylated transcriptional activator and histone modifier inside the cell. This PPI also regulates the pathway of cancer [13–17].

Public databases and literature report more than 17 000 non-redundant PPIMs [8,18]. The improvement in data extraction and management has aided in the identification of this huge number of compounds, which have been evaluated against different protein targets using various computational techniques. The advantage of this approach is that PPIMs can bind to many types of protein interfaces including orthosteric and allosteric sites, thus are often used as a starting point for PPI-targeting drug discovery programmes compared with other drug discovery strategies [19].

In spite of the progress in PPIM drug discovery, the rate of success to find lead compounds in high-throughput screening techniques using these synthetic small molecules remains quite low. We have compiled a collection of known PPI inhibitors and used this dataset in machine learning methods. We present support vector machine (SVM)-based classifier prediction based web server with 10 standard physico-chemical properties/descriptors to build the optimal models for known PPIs like Mdm2/P53, Bcl2/Bak and c-Myc/Max [20]. The predicted SVM scores of training/testing datasets of Mdm2/P53 and Bcl2/Bak were compared with IC50 values and docking scores. Finally, the screened small chemicals from a large independent dataset from National Cancer Institute (NCI) were subjected to docking studies to find out a relationship between high and random predicted SVM scores with AutoDock Vina scores.

## 2. Material and methods

### 2.1. Data collection for various datasets

#### 2.1.1. Cross-validation dataset

The data of distinct small molecules (inhibitors) for three PPIs, Mdm2/P53, Bcl2/Bak and c-Myc/Max, were downloaded from TIMBAL and PubChem database. About 80% of total positive dataset was used as positive set for fivefold cross-validation, i.e. training/testing data. The positive datasets of Mdm2/P53, Bcl2/Bak and c-Myc/Max consisted of 250, 735 and 15 small molecules, respectively. PubChem BioAssay structure clustering (https://pubchem.ncbi.nlm.nih.gov/assay/assay.cgi?p=clustering) tool was used to make sure that the chemicals in training and testing set for all the three datasets are non-redundant. In the case of Mdm2/P53 and Bcl2/Bak, the negative sets were prepared by choosing 1040 random chemicals from PubChem and adding the other two positive set of PPIMs. For example, Bcl2/Bak and c-Myc/Max positive sets were included in Mdm2/P53 negative set along with 1040 random chemicals. In the case of c-Myc/Max, there were only 15 PPIMs in the positive set, so we have only taken random small chemical dataset as the negative set which is equivalent to 10 times the positive set. Therefore, the negative datasets of three PPIs (Mdm2/P53, Bcl2/Bak and c-Myc/Max) became 1790, 1305 and 150 molecules, respectively. The positive and negative set values are shown in electronic supplementary material, table S1*a*, and were further divided into five equal parts for fivefold cross-validation technique.

#### 2.1.2. Non-redundant chemical datasets based on structural similarity

PubChem BioAssay structure clustering tool was used to create the non-redundant positive datasets based on 90% and 80% structural similarity of the small chemical PPIMs. The positive datasets of 90% and of 80% Mdm2/P53 dataset reduced to 75 and 40 small chemicals, respectively and for Bcl2/Bak dataset were 185 and 100, respectively. For Mdm2/P53 and Bcl2/Bak, 1 : 1 (positive: negative) ratio datasets were created separately considering 0.99, 0.90 and 0.80 structural similarity threshold. Myc/Max dataset was not used in this study, due its small size.

#### 2.1.3. Blind dataset

Remaining 20% of the positive sets for Mdm2/P53, Bcl2/Bak and c-Myc/Max including 30, 100 and 5 PPIMs, respectively that were obtained from TIMBAL were used as blind dataset. These sets were not used in training and testing. The negative blind sets were created in two subsets for each PPI complex, i.e. 1 : 1 (P : N) and 1 : 10 (P : N) randomly from PubChem (electronic supplementary material, table S1*b*).

#### 2.1.4. Independent (large) dataset

NCI database that was released in May 2012 consisting of 265 242 structures was processed and finally 216 103 structures were used as a large independent dataset. The structures that did not have xlogP3 value were removed.

#### 2.1.5. 2P2I dataset

2P2I positive dataset consisting of 40 PPIMs was used for comparison study [21].

#### 2.1.6. Dataset for drug-like molecule similarity

All the positive datasets of Mdm2/P53, Bcl2/Bak and c-Myc/Max consisting of 250, 735 and 15 small molecules were used as a database in SDF two-dimensional format for drug-like similarity search algorithm.

All the datasets used in this study are available in ‘about page’ of PPIMpred at http://bicresources.jcbose.ac.in/ssaha4/PPIMpred/about.php.

### 2.2. Machine learning techniques

#### 2.2.1. Feature selection as molecular descriptors

We compiled the physico-chemical properties of both positive and negative datasets of all the three PPI complexes from PubChem. Initially, 18 descriptors of each chemical structure were extracted for feature selection. Student *t*-test was performed in Mdm2/P53 and Bcl2/Bak datasets for feature selection and finally 10 descriptors with a *p*-value of less than 0.05 were selected [22].

#### 2.2.2. Support vector machine

The SVM^light^ package was used to classify the PPIM against the three PPI complexes [23]. The different kernels of SVM, namely, (i) linear, (ii) polynomial and (iii) radial basis function (RBF) kernel, were used for developing the models.

#### 2.2.3. Naive Bayes and Random Forest

We also used two other machine learning techniques, namely Naive Bayes and Random Forest methods by Weka tool [24].

### 2.3. Comparison of IC50 value with predicted support vector machine score of known protein–protein interaction modulators

The positive training set chemicals from Mdm2/P53 and Bcl2/Bak were mapped to ChEMBL database [25]. Many of them were found to have IC50 values for specific PPIs. The IC50 values were extracted and converted to log scale and then compared with SVM scores. There was no reasonable report of known IC50 value of chemicals against Myc/Max.

### 2.4. Performance measures

The fivefold cross-validation technique was used to analyse the performance of different classifiers. The dataset of small chemicals of three mentioned PPIs (Mdm2/P53, Bcl2/Bak and c-Myc/Max) was divided randomly into five subsets. The machine learning technique classifiers were trained on four sets and performance was measured on the fifth set. The process was continued up to five times so that each set could be used for testing. The average performance of classifiers on five sets is considered to be the final performance. The threshold-dependent parameters sensitivity, specificity, accuracy, precision (PPV), F1 score were used. Also, threshold-independent parameter area under receiver operating characteristic (ROC) curve was also measured.

$$\begin{array}{rl} \text{Sensitivity} & =\frac{TP}{TP+FN}\\ \text{Specificity} & =\frac{TN}{TN+FP}\\ \text{Accuracy} & =\frac{TP+TN}{TP+FN+TN+FP}\\ \text{PPV} & =\frac{TP}{TP+FP}\\ \text{F1} & =\frac{2TP}{2TP+FP+FN}. \end{array}$$

### 2.5. Confidence measure

The predicted SVM scores of known positive and negative sets were plotted in a histogram, and an unknown predicted query score was used to validate it from these plots by computing the area under the curve (AUC). If a predicted SVM score has a higher AUC in the positive plot then the confidence of the prediction to be positive PPIM will be higher (details in electronic supplementary material, figure S1).

### 2.6. Randomized trial

The randomized datasets of Mdm2/P53 and Bcl2/Bak were prepared using the 1 : 1 (positive:negative) datasets of 0.99 chemical structural similarity. The positive and negative labels were assigned randomly. These randomized datasets were used for fivefold cross-validation using SVM-based method and threshold-dependent and -independent measures were computed.

### 2.7. Structural similarity based method

We implemented a similarity searching method to find similar chemicals against user query input (a structure or a mol file). ChemmineR package was used [26,27], where a function cmp.similarity function computes the atom pair similarity between two compounds using the Tanimoto coefficient as similarity measure. The function returned a data frame where the rows were sorted by the Tanimoto similarity score (best to worst). It is the proportion of the atom pairs shared among two compounds divided by their union. The formula of Tanimoto similarity is

$$\text{Tanimoto coefficient}=\frac{c}{a+b+c}.$$

The variable *c* is the number of atom pairs common in both compounds, where *a* and *b* are the numbers of their unique atom pairs.

### 2.8. Docking studies

Docking was performed using AutoDock Vina [28]. Three-dimensional structures of small molecules from NCI dataset were taken as ligands and the crystal structures of Mdm2 (PDB id: 1YCR), Bcl2 (PDB id: 2XA0) and Myc (PDB id: 1NKP) were taken as receptors (targets). Ligands and receptors were prepared using AutoDockTools for docking [29]. Docking study was focused on the small molecule sets with highest, lowest and randomly selected SVM predicted scores. The known PPIMs from training/testing datasets against Mdm2/P53 and Bcl2/Bak were also subjected to docking studies.

## 3. Results and discussion

### 3.1. Selection of protein–protein interaction complex

In this study, three PPI targets, namely, Mdm2/P53, Bcl2/Bak and c-Myc/Max, were considered. The data distribution of PPIs from TIMBAL and specific chemical targets are shown through a pie chart in electronic supplementary material, figure S2, which clearly shows that Mdm2/P53 and Bcl2/Bak were the top PPI hits of known PPIMs. Although Myc/Max has few hits, it was considered due to its biological significance in disease biology. Besides these, PDB structures of these complexes were available.

### 3.2. Features selection

Eighteen physico-chemical features were extracted for each chemical in the positive and negative PPIM sets. A *t*-test was performed to filter out the non-significant features in targeting three PPI complexes, Mdm2/P53, Bcl2/Bak and c-Myc/Max, as shown in electronic supplementary material, table S2*a*–*c*. The same set of 10 descriptors including (i) molecular weight, (ii) xlogp3, (iii) hydrogen bond donor count, (iv) rotatable bond count, (v) topological polar surface area, (vi) heavy atom count, (vii) complexity, (viii) defined atom stereocentre count, (ix) defined bond stereocentre count, and (x) covalently bonded unit count were shown to be significant with *p*-value of less than 0.05 in Mdm2/P53 and Bcl2/Bak datasets. Thus for further analysis, we selected these 10 descriptors for evaluation of the machine-learning techniques. Although the trend was different in c-Myc/Max dataset, probably due to a small number of positive examples (*n* = 15).

### 3.3. Performance of support vector machine, Naive Bayes and Random Forest using fivefold cross-validation

Threshold-dependent and independent measures were used to classify the PPIMs against three PPI complexes (Mdm2/P53, Bcl2/Bak and c-Myc/Max) using SVM, Naive Bayes and Random Forest as shown in table 1*a*–*c*. It is important to remember that in Mdm2/P53 dataset there were 250 positive and 1790 negative (random, Myc/Max positive and Bcl2/Bak positives) small chemicals, whereas in Bcl2/Bak dataset there were 735 positives and 1305 negatives (random, Myc/Max and Mdm2/P53) and in Myc/Max there were only 15 positives and 150 negatives (random). Among three SVM kernels, RBF was performing better in Mdm2/P53 and Bcl2/Bak datasets as shown in electronic supplementary material, table S3*a*–*c*, and ROC plots in electronic supplementary material, figure S3*a*–*c*, and the density plots of positive and negative training sets in electronic supplementary material, figure S4*a*–*c*. Although Random Forest performed best in terms of overall accuracy and AUC, the sensitivity was higher in SVM RBF kernel in all the three sets.

**Table 1:** (*a*) Comparison of performance on Mdm2/P53 (1 : 7) testing dataset (fivefold cross-validation) using three different kernels of SVM (linear, polynomial and radial basis function), Naive Bayes and Random Forest method. (*b*) Comparison of performance on Bcl2/Bak (1 : 2) testing dataset (fivefold cross-validation) using three different kernels of SVM (linear, polynomial and radial basis function), Naive Bayes and Random Forest method. (*c*) Comparison of performance on c-Myc/Max (1 : 10) testing dataset (fivefold cross-validation) using three different kernels of SVM (linear, polynomial and radial basis function), Naive Bayes and Random Forest method.

methods	sensitivity	specificity	accuracy	F1 score	PPV	AUC
(*a*)						
SVM linear	0.68	0.71	0.70	0.36	0.41	0.77
*c* = 1, *j* = 1						
SVM poly	0.64	0.60	0.61	0.35	0.32	0.63
*d* = 1, *c* = 1, *j* = 3						
SVM RBF	0.83	0.82	0.83	0.45	0.57	0.88
*g* = 0.0001, *c* = 10, *j* = 8						
Naive Bayes	0.16	0.97	0.87	0.22	0.39	0.83
Random forest	0.69	0.99	0.95	0.77	0.88	0.93
(*b*)						
SVM linear	0.73	0.60	0.65	0.67	0.62	0.69
*c* = 1, *j* = 2						
SVM poly	0.60	0.49	0.53	0.49	0.48	0.61
*d* = 1, *c* = 1, *j* = 3						
SVM RBF	0.86	0.75	0.79	0.72	0.77	0.83
*g* = 0.0001, *c* = 1, *j* = 2						
Naive Bayes	0.70	0.87	0.81	0.73	0.76	0.87
Random forest	0.87	0.94	0.92	0.88	0.90	0.95
(*c*)						
SVM linear	0.80	0.93	0.92	0.60	0.65	0.89
*c* = 1, *j* = 1						
SVM poly	0.8	0.90	0.89	0.47	0.58	0.89
*d* = 1, *c* = 10, *j* = 3						
SVM RBF	0.87	0.91	0.90	0.50	0.63	0.91
*g* = 0.0001, c = 1, *j* = 1						
Naive Bayes	0.67	0.95	0.93	0.63	0.59	0.86
Random forest	0.4	0.99	0.94	0.55	0.86	0.89

**Figure 1.**
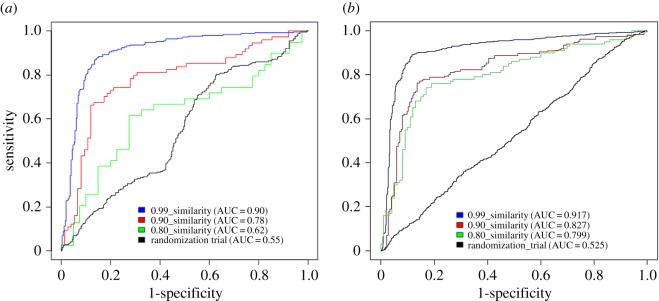
(*a*) The ROC plot for Mdm2/P53 1 : 1 (P : N) dataset with 0.99 similarity (blue), 0.90 similarity (red), 0.80 similarity (green) and randomization trial (black). (*b*) The ROC plot for Bcl2/Bak 1 : 1 (P : N) dataset with 0.99 similarity (blue), 0.90 similarity (red), 0.80 similarity (green) and randomization trial (black).

In addition, the positive and negative datasets with 1 : 1 (positive: negative) of 0.99, 0.90 and 0.80 structural similarity and with randomized dataset for Mdm2/P53 and Bcl2/Bak were used for training and testing using SVM RBF kernel. The ROC plots in figure 1*a*,*b* show that the AUC was maximum in 0.99 structural similarity for both Mdm2/P53 and Bcl2/Bak. The AUC values were decreasing in the case of 0.90 and 0.80 in Mdm2/P53 and Bcl2/Bak datasets. These plots show that the randomized dataset AUC values were 0.55 and 0.52 for Mdm2/P53 and Bcl2/Bak datasets, respectively. Similar trend was observed in the other dataset where number of negative examples were higher than positive set (electronic supplementary material, figure S5*a*,*b* and tables S4*a*,*b*, S5*a*,*b* and S6*a*,*b*).

The small chemical ligands considered in positive training sets of the two PPIs (Mdm2/P53, Bcl2/Bak) were mapped to the ChEMBL database and a structure–activity relationship parameter, i.e. IC50 values, was studied. The IC50 values for Mdm2/P53 and Bcl2/Bak were taken and a comparison was drawn among the log transformed IC50 values and predicted SVM scores, as shown in electronic supplementary material, figure S6*a*,*b*. In the case of Mdm2/P53, about 89% chemicals have SVM score above 0.5 with reasonable IC50 values. Similar trend was observed for Bcl2/Bak, of about 82% chemicals having SVM score above 0.5 with reasonable IC50 values.

To establish the confidence of effectiveness of the method, the comparison among docking scores, i.e. binding free energy values and SVM scores of the training set, was also drawn for Mdm2/P53 and Bcl2/Bak. The plots are available in electronic supplementary material, figure S7*a*,*b*. Interestingly, it is found that about 89% of chemicals for Mdm2/P53 and about 82% chemicals for Bcl2/Bak have SVM scores above 0.5 with docking scores less than −7 kcal mol^−1^. The docking studies showed that the small chemicals from training/testing set of Mdm2/P53 bind to Mdm2 at the P53 binding site (as shown in electronic supplementary material, figure S8*a*). Similarly, it was observed that the training set PPIMs of Bcl2/Bak bind to Bcl2 on the binding site of Bak (as shown in electronic supplementary material, figure S8*b*).

### 3.4. Assessment on blind dataset using support vector machine-based method

The accuracies of the blind set in Mdm2/P53 in two different ratios (1 : 1 and 1 : 10) were 84% and 64% (electronic supplementary material, table S7*a*,*b*). In 1 : 10 dataset, the specificity decreases; thus the overall accuracy was low. However, the overall sensitivity in both the sets, i.e. 1 : 1 and 1 : 10, remains more or less the same. Thus, the SVM model developed using fivefold cross-validation with only 10 descriptors is able to predict the unknown set not used in training and testing the models with reasonable accuracy. The overall accuracies in blind datasets (1 : 1 and 1 : 10) in Bcl2/Bak were 66% and 63% (electronic supplementary material, table S8*a*,*b*) with higher sensitivities. A similar trend was observed in c-Myc/Max blind dataset assessment (electronic supplementary material, table S9*a*,*b*).

### 3.5. Prediction and validation of unknown large National Cancer Institute small chemical dataset

NCI database consisting of more than 250 000 small chemicals was checked in the proposed SVM models of Mdm2/P53, Bcl2/Bak and c-Myc/Max. The predicted SVM scores above zero (to avoid false positives) of three models were plotted using histogram density plots function and a significant threshold was marked as shown in electronic supplementary material, figure S9*a*–*c*. A highly significant set of PPIMs were selected from these plots by choosing the threshold value (for Mdm2/P53 over 1.9, for Bcl2/Bak over 1.4 and for c-Myc/Max over 1.7) in the right-tail of the *x*-axis as shown in electronic supplementary material, table S10. Interestingly, the inhibitors predicted against three complexes (473, 466 and 232 for Mdm2/P53, Bcl2/Bak and c-Myc/Max, respectively) are mutually exclusive as there were no overlaps among them (electronic supplementary material, figure S10 and table S11).

**Figure 2.**
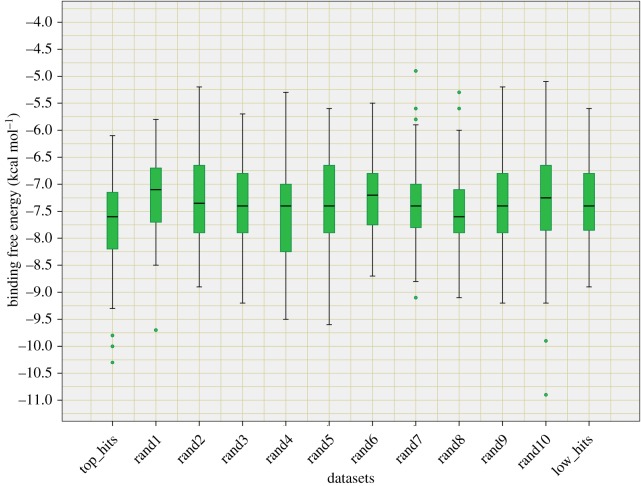
Box plot showing the binding free energy of top hits, low hits and random hits of Bcl2/Bak.

The top hits from SVM predicted PPIMs for all the three complexes were further used for *in silico* docking by AutoDock Vina and a significance test was performed by comparing with random and low scoring SVM scores of NCI chemicals set. The docking results were obtained in the form of binding free energies for an interaction of the small molecules with three protein targets (Mdm2, Bcl2 and Myc). The box plot of AutoDock scores binned in three different SVM predicted NCI molecule scores, i.e. top hit (*n* = 60), low hits (*n* = 60) and 10 random hits (*n* = 60*10), for Bcl2 is shown in figure 2 and for Mdm2 and Myc are shown in electronic supplementary material, figures S11*a* and S12*a* and the electronic supplementary material, table S12 shows the values obtained from boxplot. Similarly, the distribution plot of AutoDock scores in three different bins was also plotted (electronic supplementary material, figures S11*b*, S12*b* and S13). The names of the 60 top NCI small chemical hits against three targets used in this study are available in electronic supplementary material, table S13*a*–*c*. The significance *t*-Test *p*-values for Bcl2, Mdm2 and Myc were 0.11, 0.56 and 0.14. Although these results were not significant at *p*-value of 0.05 level, at least in Bcl2 and in Myc it was significant at 0.1 level (*p*-value < 0.1). In summary, we observed that SVM scores predicted in top hits are better in comparison with random based on docking studies.

### 3.6. Comparison with another method

There is no server dedicated for predicting specific PPIMs against Mdm2/P53, Bcl2/Bak and c-Myc/Max. However, there is a server, 2P2I_HUNTER_, which can predict whether a chemical can be an orthosteric PPIM. In this tool, SVM with a radial Gaussian basis kernel was used to train 40 non-redundant small molecules as a positive set and 1018 compounds as random (decoy) set. Within 40 PPIMs, there were seven inhibitors for Mdm2/P53, 10 inhibitors for Bcl2/Bak and none for c-Myc/Max (the Venn diagram is shown in electronic supplementary material, figure S14*a*, and details in table S14). The overall performance of their optimized model was a sensitivity of 63% and specificity of 100%. We have used this dataset in three of our optimized models specific for Mdm2/P53, Bcl2/Bak and c-Myc/Max. At a threshold value of 0.8, and using 40 PPIMs we observed 16 inhibitors specific for Mdm2/P53, 20 inhibitors for Bcl2/Bak and one for c-Myc/Max (shown in electronic supplementary material, tables S15 and S16). Interestingly, all the seven reported inhibitors for Mdm2/P53, 10 reported inhibitors for Bcl2/Bak among 40 were picked up by SVM models based on radial basis kernel (RBF) (the Venn diagram is shown in electronic supplementary material, figure S14*b*). 2P2I hunter used 11 descriptors, where as we used 10 descriptors, with five common descriptors (logP, molecular weight, topological surface area, hydrogen donor and rotation bond).

### 3.7. Web server

**Figure 3.**
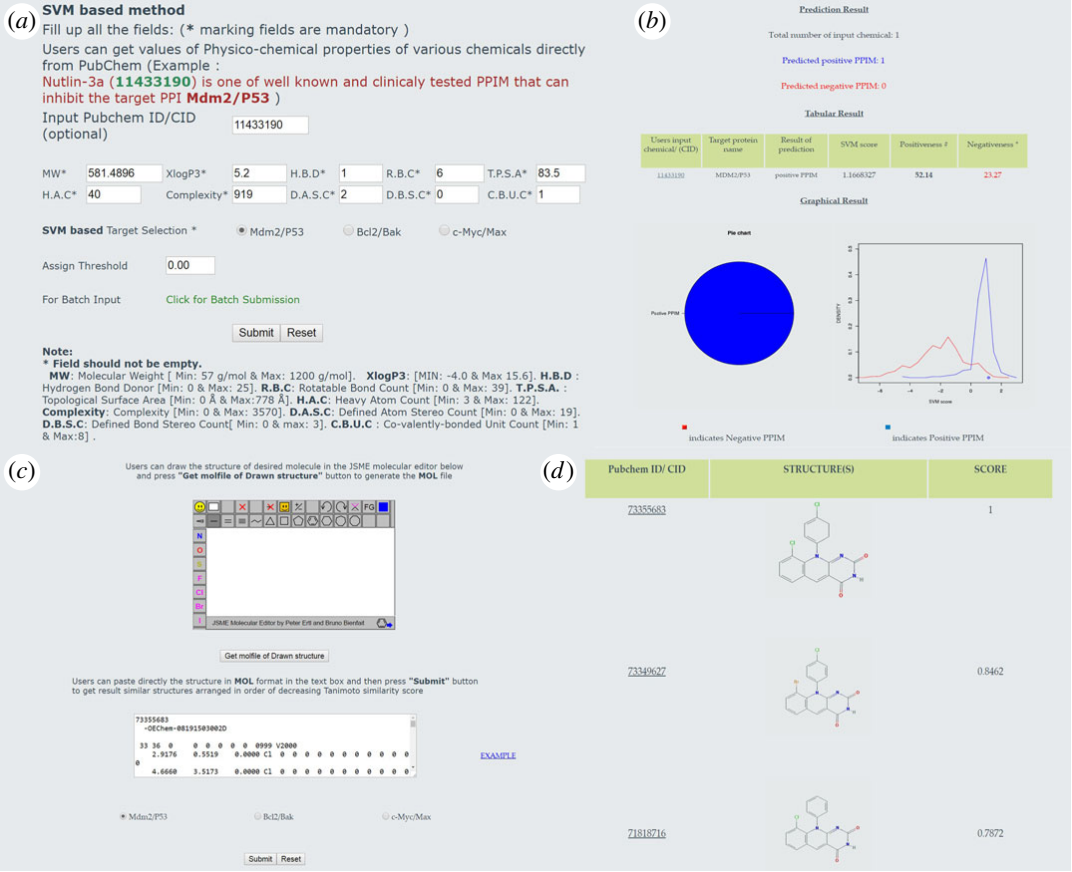
(*a*) The home page consisting of submission form for molecular descriptors, target selection and threshold value selection. (*b*) Result page of prediction shows ‘prediction result’, ‘tabular result’ and ‘graphical result’. (*c*) Similarity search page where users can input a molecule either by drawing using JME editor or by pasting MOL 2D format file. (*d*) Similarity search result shows the list of compounds similar to the query structure.

Three different SVM-based models were used to develop PPIMpred web server for classification of PPIMs targeting Mdm2/P53, Bcl2/Bak and c-Myc/Max PPIs. There are two separate input pages, namely ‘molecule search’ and ‘similarity search’, in the web server for end users. The molecule search has two options: single molecule search and batch input as shown in figure 3*a*. Single molecule search option allows users to provide molecular descriptors, target selection (Mdm2/P53, Bcl2/Bak and c-Myc/Max) and defining threshold value. The molecular descriptors include (i) molecular weight, (ii) XLogP3, (iii) hydrogen bond donor count, (iv) rotatable bond count, (v) topological polar surface area, (vi) heavy atom count, (vii) complexity, (viii) defined atom stereocentre count, (ix) defined bond stereocentre count, and (x) covalently bonded unit count. Batch input option allows users to upload a file containing the above 10 descriptor information of a list of molecules in a comma delimited format (.csv file). Similarity search page allows users to draw desired chemical structure using JME tool or directly paste MOL file of the desired chemical to the text area and after that choosing a target PPI from radio button as shown in figure 3*c*.

The output from PPIMpred for molecular search option has three sections: (i) prediction result, (ii) tabular result, and (iii) graphical result as shown figure 3*b*. In the prediction result section users get a number of positive and negative PPIMs, and the tabular section displays SVM score and prediction confidence measured in terms of positiveness and negativeness. In the case of batch upload option, result summary is shown in the graphical section as pie charts. In addition to it, in similarity search option, the server provides similar structures used for known PPIMs ranked based on Tanimoto similarity score of chemical input query. Each hit from PubChem Id is hyperlinked to PubChem CID [18] for further information as shown in figure 3*d*.

The clinically tested PPIMs for the target PPIs were already present in the positive training/testing set. They were found to have an SVM score above 0.5; Nutlin-3a (Mdm2/P53) has an SVM score of 1.17, ABT-263 (Bcl2/Bak) has an SVM score of 1.01 and GX15-070(Bcl2/Bak) has an SVM score of 0.56 (shown in electronic supplementary material, table S17). The position of the chemicals in density plot is shown in electronic supplementary material, figure S15*a*,*b*.

## 4. Conclusion

Focus of this study was to develop a user-friendly, and publicly accessible web server to identify lead PPIM molecules *in silico* for three clinically relevant protein complexes, because experimental screening of the huge chemical spaces is a relentless task. Our proposed PPIMpred web server can be useful for high-throughput screening of large chemical datasets for lead recognition. Machine-learning methods were used for classification of the data. SVM with three different kernels (linear, polynomial and RBF) was used to find the optimal model for classification. Naive Bayes and Random Forest methods of machine learning were also performed. In addition to categorical classification, it also gives hints of structural similarity with known drug-like molecules for further insights. PPIMpred has two separate search pages for finding predicted PPIMs and for similarity search. For making the search user-friendly, a batch input option is also present. The comparison analysis of known chemicals in the training and testing sets against Mdm2/P53 and Bcl2/Bak predicted SVM score with IC50 and predicted SVM score with docking score were performed. For further validation of the method, docking study was also performed on the top hits, low scoring hits as well as random hits obtained from validation of independent set. The docking results are analysed using statistical methods like boxplot and density plot. The docking study shows the top scoring molecules to be better modulators for the PPIs. The screening of a large chemical dataset of NCI gives exclusive hits for the three PPIs that are focused in our study, namely, Mdm2/P53, Bcl2/Bak and c-Myc/Max. These hits can be further subjected to *in silico* as well as experimental approaches for identification of lead candidates.

## Data accessibility

All supporting information like tables and figures is included in the electronic supplementary material.
